# Targeting children of substance-using parents with the community-based group intervention TRAMPOLINE: A randomised controlled trial - design, evaluation, recruitment issues

**DOI:** 10.1186/1471-2458-12-223

**Published:** 2012-03-22

**Authors:** Sonja Bröning, Annika Wiedow, Lutz Wartberg, Sylvia Ruths, Andrea Haevelmann, Sally-Sophie Kindermann, Diana Moesgen, Ines Schaunig-Busch, Michael Klein, Rainer Thomasius

**Affiliations:** 1 University Medical Centre Hamburg-Eppendorf, Centre for Psychosocial Medicine, German Centre for Addiction Research in Childhood and Adolescence, Martinistraße 52, D-20246 Hamburg, Germany; 2 Catholic University of Applied Sciences Nordrhein-Westfalen, German Institute for Addiction and Prevention Research, Wörthstraße 10, D-50668 Köln, Germany

**Keywords:** Selective prevention, Children of substance abusers, Prevention programmes, Substance-affected families, RCT, At-risk children

## Abstract

**Background:**

Children of substance-abusing parents are at risk for developing psychosocial development problems. In Germany it is estimated that approx. 2.65 million children are affected by parental substance abuse or dependence. Only ten percent of them receive treatment when parents are treated. To date, no evaluated programme for children from substance-affected families exists in Germany. The study described in this protocol is designed to test the effectiveness of the group programme TRAMPOLINE for children aged 8-12 years with at least one substance-abusing or -dependent caregiver. The intervention is specifically geared to issues and needs of children from substance-affected families.

**Methods/Design:**

The effectiveness of the manualised nine-session group programme TRAMPOLINE is tested among N = 218 children from substance-affected families in a multicentre randomised controlled trial. Outpatient counselling facilities across the nation from different settings (rural/urban, Northern/Southern/Eastern/Western regions of the country) will deliver the interventions, as they hold the primary access to the target group in Germany. The control condition is a group programme with the same duration that is not addiction-specific. We expect that participants in the intervention condition will show a significant improvement in the use of adaptive coping strategies (in general and within the family) compared to the control condition as a direct result of the intervention. Data is collected shortly before and after as well as six months after the intervention.

**Discussion:**

In Germany, the study presented here is the first to develop and evaluate a programme for children of substance-abusing parents. Limitations and strengths are discussed with a special focus on recruitment challenges as they appear to be the most potent threat to feasibility in the difficult-to-access target group at hand (Trial registration: ISRCTN81470784).

## Background

The negative impact of parental substance use on children has been documented by a multitude of studies and reviews, especially for children of alcoholics. It includes physical, psychological and cognitive consequences for children's development. Children and adolescents affected by parental drug use show higher rates of externalizing and internalizing problems such as anxiety disorders and depression [1,2], social behaviour disorders [3,4], or hyperactivity disorders [4,5]. With regard to substance use problems, their records more often show an early onset of substance consumption [6,7], earlier drunkenness experiences [8], increased binge drinking rates [9] and an elevated risk for developing substance use disorders at a younger age than comparable peers [10]. Approximately 33 to 40 percent of all children with a substance-using parent will develop a substance use disorder themselves [11]. Substance problems are transmitted to the next generation via several pathways, especially genetic disposition [12,13], exposure to drugs in utero, behavioral and cognitive processes [14,15]. Family environmental characteristics such as problematic family relationships, family conflict or absence of supportive parenting [16] also play an important part. Children from substance-affected families often experience physical violence, emotional abuse, or neglect [17]. Paradoxically, at the same time they often acquire the same positive expectations about the effects of substance consumption that their substance-abusing parent exhibits [18,19]. Learning from the example substance-dependent parents set, children later come to use substances as maladaptive coping strategy in stressful and difficult times [20-22]. Behavioural consequences in childhood and adolescence are gender-specific: while females lean toward withdrawal and social isolation, males often show antisocial behaviour [17]. In general, male children seem to have more difficulties with compensating for family problems resulting from parental alcohol or drug use. They more often develop attention deficit problems, anxiety, depression and early alcohol problems [23]. However, not all children from substance-affected homes show maladaptive functioning. In fact, about one third of them exhibit no psychosocial problems in spite of the adverse circumstances associated with parental substance use [24,25]. These persons are often called "resilient".

Research on resilient children from substance-affected homes shows that these children are different from less well-adapted ones in several ways: First, they are more likely to have social resources outside of their parents such as caring relatives or friends [26]. Second, they may be less exposed to parental alcohol or drug problems, or their substance-affected parent may have undergone a successful, abstinence-oriented treatment [26]. Third, they exhibit personality traits that contribute to their resilience such as high stress resistance, good adaptation skills in new situations and high self-efficacy [27]. Wolin and Wolin [28] identified seven characteristics of resilient children of substance-abusing parents: Insight, autonomy, robust relationships, initiative, creativity, humour and morality. With regard to interventions, strengthening the resilience of these children-at-risk is often viewed as an important goal. Research on the efficacy of interventions designed specifically for children from substance-affected families has mainly focused on two kindes of interventions: family-focused prevention efforts aimed at increasing supportive and nurturing parenting [29-31] and (mainly school-based) peer group programmes that draw on the effects of positive peer influence and mutual support [32-34]. The majority of these studies have demonstrated positive programme effects in the area of coping skills, programme-related knowledge and social behaviour [35]. While these results seem promising, research on selective prevention programmes targeting children of substance abusers still remains rather scarce, especially outside of the United States.

In Germany, it is estimated that approx. 2.65 million children are affected by parental alcohol abuse or depencence until they come of age [36]. A more recent study concludes that five to six million youth under the age of twenty have at least one parent with alcohol problems [37]. It is likely that an additional large number of unreported cases exist [38]. Germany holds a highly differentiated addiction care system for alcohol and drug users that includes low-threshold offers such as outreach work, but also outpatient treatment centres, inpatient treatment services in psychiatric clinics as well as inpatient rehabilitation treatment. Outpatient treatment facilities are widespread, in urban as well as rural areas, and provide low-threshold and cost-free counselling that is funded by the communities, the federal states and/or big welfare organisations. Staff members within these centres have diverse professional backgrounds ranging from social workers, pedagogues, psychologists and psychotherapists to medical staff. Due to their different roots, institutions operate with a variety of philosophies and therapeutical concepts. They can refer clients in need of more intensive care into either outpatient (by a physician) or inpatient substance disorder treatment (detoxification, withdrawal treatment) financed by health insurance. Outpatient walk-in and day clinics specialised in alcohol and drug treatment also exist in larger medical centres. Patients may also enter all of these medical centres directly, without referral. An ideally seamlessly adjoining long-term medical rehabilitation treatment is financed by pension insurance. Therapeutic approaches are usually multimodal, combining different psychotherapeutic approaches and using cognitive-behavioral, behavioral-therapeutic, psychodynamic and systemic or family therapeutic elements. These are applied in a diagnosis-oriented treatment and offered as individual or group therapy. Thus, in the vast majority of all cases, professional assistance for persons with substance-related problems is available and covered by insurance. Often, however, the situation of children is not taken into account when a parent enters the German drug aid system. Even though current data do not exist, statistics from 1998 show that when parents receive addiction treatment or counselling, only ten percent of all children are also treated [39].

Recently, the abovementioned findings on the developmental risks for children of substance-affected parents have spurred diverse local efforts to help, especially in out-patient settings. Arenz-Greiving and Kober [40] estimated in 2007 that about 40-50 interventions for this target group exist in German outpatient centres. These interventions included a wide variety of concepts and settings such as individual counselling, group programmes, parent work, play groups, holiday camps and many others. The authors also found a few inpatient programmes for children of parents receiving alcohol or drug treatment in medical centres. Other than in the United States, for example, schools do not offer selective programmes for children of parents with drug problems for fear of stigmatising them. Taking into account the large number of affected children, however, help for children of parents with alcohol or drug problems is still rare in Germany. In addition, programme deliverers frequently face substantial recruitment challenges that will be detailed in the next section. Many existing programmes are not ongoing, but remain project-type efforts. This is mainly due to funding issues: interventions for children from substance-affected families are not covered by German health insurance, but are funded - often for a limited period of time only - by the communities, the federal states, or regional initiatives. All in all, there are several programmes for these at-risk children, but programme delivery is rather unsystematic due to the mulitfaceted and heterogeneous help system for affected families. Moreover, previous efforts are not evidence-based.

Our own study conducted in 2009 identified 48 outpatient counselling centres that offered prevention programmes for children of substance-using parents [41]. About half of these children were 8-12 years old. The intervening centres were most often part of either the addiction aid ("Suchthilfe") or from the the area of youth welfare aid ("Jugendhilfe") or from a combined form. With regard to the format of these interventions, group programmes were indicated most frequently (81.8% of the institutions) followed by individual counselling (61.4%) and family counselling (43.2%). Working with parents alongside the children's programme was also reported by 59.1% of the institutions. In recruiting children for the interventions, about two thirds of them were recruited by their parents who were receiving counselling in the centre itself or by other institutions within the centres network. Only few cases were referred into the programme by school or daycare centres, even though it can be assumed that staff in these institutions should often be able to identify children with substance-abusing parents. The majority of all group programmes (80%) were open and continuous, i.e. without any defined starting and ending points. They were also neither manualised nor evaluated [41]. Therefore, the study at hand will be the first to provide data on the effectiveness of a manualised group programme for children from substance-affected families in Germany.

### Objectives and hypotheses

The objective of our study is to assess the effectiveness of the community-based group programme TRAMPOLINE for children aged 8-12 years with at least one substance-abusing parent. The effectiveness of the intervention will be tested two weeks and six months after the intervention. We expect that as a direct result of the intervention, participants in the intervention condition will show a significant improvement in the use of constructive coping strategies (in general and within the family) compared to the control condition. We furthermore hypothesise that this effect will continue or become stronger in the 6-month follow-up data collection point (primary hypothesis). We also expect that exposure to the intervention will lead to other significant improvements in the intervention participants, especially in the area of psychological stress and addiction-related knowledge compared with the control condition (secondary hypothesis). Here also, we expect that these effects will be demonstrated directly after the intervention and in the 6-month follow-up measurement. For the parents, TRAMPOLINE offers an optional two-session accompanying programme. We will explore if these parent modules are also effective in improving caregivers' sensitivity about their children's needs within the substance-abusing family, caregivers' parenting skills, caregivers' feelings of self-competence with regard to parenting skills and caregivers' willingness to accept help from the outside. Another goal of our study is to explore the age- and gender-specific effectiveness of the programme. Given the fairly wide age range of participants and the above-mentioned gender differences in reacting to family problems, programmes like TRAMPOLINE need to be problem-specific and allow for these differences at the same time. Also we will explore regional effects such as differences in urban or rural settings on program success. We further aim at gathering more insight with exploratory analyses on characteristics of our target group, i.e. children from substance-affected families, by applying a broad battery of measurements, because this population is still vastly understudied in Germany. With regard to process evaluation, we will examine whether the intervention is delivered in time and according to defined standards, if the manual is adhered to and whether the programme meets the expectations of caregivers, parents and involved children.

Finally, we are interested in exploring strategies with which programme deliverers meet recruitment challenges. These challenges are manifold: (1) Frequently, affected parents deny their substance use problem. Even if their spouse or other relatives would like to see the child in such a programme, the substance-abusing parent will object to their participation on the grounds that family and parenting are private and should be kept so. (2) Due to the negative consequences of their substance abuse itself, affected parents and their spouses who realise the problem often experience *guilt or shame*. They also often fear the consequence of the help system intervening if their problems become known, up to the point of fearing that their children will be taken out of the family and placed with foster caregivers. Thus, the issue is frequently treated as a "taboo" within the family, meaning it is not addressed in conversation,and children are not allowed to speak about the problem outside of the family. For many parents, having a child talk about their "problem" in a public health setting is a very high threshold request. (3) Families often face *transportation and organisation difficulties*, especially in rural areas. If families have to drive extended distances to participate in a programme, this will lower their motivation. For many families a weekly programme in itself can pose a substantial problem of daily routine organisation, especially if this routine is already difficult to handle due to substance problems. Families with lower socioeconomic status and few resources are known to be a difficult target group for psychological interventions. (4) Finally, recruitment challenges are further heightened by the *nature of the research project *itself. Feelings of distrust toward research and randomisation, negative perceptions and fear of a lack of anonymity and of being "x-rayed" are to be expected during data collection and during the intervention itself. During the project, we developed detailed recruitment strategy recommendations to address these possible barriers to the feasibility of conducting a group programme for children from these families. We will specify these in the discussion section.

## Methods/Design

### Trial design

The effectiveness of the nine-session group programme TRAMPOLINE for children from substance-affected families will be tested in a multicentre randomised controlled trial. Out-patient counselling facilities across the nation were chosen as project facilities since they currently have primary access to the target group in Germany. In total, 27 facilities from different settings (rural/urban, Northern/Southern/Eastern/Western regions of the country) deliver interventions. The group programme TRAMPOLINE was designed and manualised in 2008/2009 during the first phase of the 3.5-year project. It is specifically geared to the issues and needs of children and parents in substance-affected families. The control condition was originally planned as a "Treatment as usual (TAU)" group, until it became clear that TAU varies strongly between facilities due to their heterogeneous approaches and is in many cases hardly comparable to a structured group programme (e.g., individual counselling sessions over many months). Thus, to ensure comparability, we designed a group programme with the same duration that is not addiction-specific (both interventions are described below) and that functions as control group setting. This will enable us to identify effects of TRAMPOLINE that go above and beyond a general group setting with effects resulting from variables such as trainer attention or social networking. We view this as quite a rigorous test, given the fact that the common design of many international studies of this kind utilises wait-list controls and thus compares "intervention" with "no intervention" [35]. At baseline N = 218 children aged 7-13 years have entered the programme. Although the programme is designed for 8 to 12 year-olds, execeptions were made after screening for children whose developmental status fits in with this age group, allowing for the occasional 7 to 13 year-old. For each project centre, our research institute randomly assigned participating children either to the intervention condition or to the control condition. At the time this manuscript is drafted, the field phase is ongoing, with baseline data collection already completed.

### Interventions

The theoretical underpinnings of the programme were derived from existing literature. In particular, TRAMPOLINE is based on the following theoretical concepts: The challenge model by Wolin and Wolin [42] posits that a child can cope more constructively with difficult situations if it interprets them as a challenge. He or she will then actively react to the situation and, from this feeling of control, as also described in Rotter [43], will eventually develop inner strength and resilience. In their transactional stress concept, Lazarus and Folkman [44] also view the individual cognitive processing of stressful situations as critical for the kind of reaction it will evoke. Hence, interpretation and appraisal are targeted at different times during the intervention. The stress-coping-support model by Velleman and Templeton [17] adds to this by postulating that stress and distress resulting from substance abuse or dependency of a family member can be positively influenced via constructive coping strategies (e.g., insight, understanding and behavior) as well as by social support from the outside. The concept of self-efficacy in Bandura's social-cognitive learning theory [45] and, again, the development of a sense of agency is important to our intervention considering the feelings of being helpless and overwhelmed with which the children in our target group struggle. Antonovsky [46] posits that a sense of coherence is a central influencing factor on health and well-being. A sense of coherence is aquired when certain stimuli or events are experienced as understandable, manageable and meaningful. As children from substance-affected families rarely discuss events in their families with outsiders, coming to see their experience as comparable with the experiences of others in similar situations can in itself contribute substantially to their sense of coherence. Saarni's [47] concept of emotional competence highlights the need for helping the target group with emotion regulation, especially in difficult situations, but also with knowledge about emotions that might come up in oneself or in the other. Tuckman's stages of group development [48,49] guide the structuring of sessions within the programme. Several goals were identified for TRAMPOLINE based on the abovementioned concepts and aligned with the hypotheses mentioned above: (1) to teach participants effective strategies for coping with stress, such as improving their emotion regulation, enhancing their problem solving skills in addiction-related and other problem situations and encouraging them to seek help in an appropriate way from significant others (2) to reduce the psychological stress for participants resulting from parental substance abuse or dependency by, for instance, breaking the taboo of not talking about addiction-related topics (3) to extend children's knowledge about alcohol and drugs, their effects on people and the consequences of substance-related disorders for affected persons and their family (4) to improve feelings of self-worth and self-efficacy and to help develop a positive concept of self. In addition, we pursued the following goals for the parent modules: (1) to sensitize parents for children's needs and for the effects parental substance use has on children (2) to improve caregivers' self-confidence with regard to parenting skills (3) to motivate parents to seek and accept further help and support in raising their children. The name "TRAMPOLINE" was chosen because it evokes positive associations in children, but also because a trampoline combines the ability to jump higher with protection and a soft landing. In this way, the programme aims at empowering participating children while at the same time providing support and a safe place to be.

In developing the group programme TRAMPOLINE, a three-step approach was chosen. In the first step, we reviewed the international literature to identify key features of successful programmes [35]. From this review, we concluded the following requirements for the programme: (1) The programme needs to address the specific challenges children from substance-affected families face, such as questions on drugs and addiction, on coping with psychological distress resulting from parental substance abuse and on handling difficult situations arising from it. (2) Successful programmes are resource-oriented and build on the strengths of participating children. (3) Programmes use a variety of didactic methods to remain fun and interesting for the children, i.e. stories, exercises, role-play, games, body awareness exercises. (4) Single-component programmes appear less effective than programmes that comprise several components, e.g., by incorporating activities for the parents. In the second step, we invited experts in the field such as counsellors and social workers to provide us with material from their previous work with children from substance-affected families. From this knowledge exchange and the results of our literature review, we drafted a first version of the TRAMPOLINE manual. This was adapted after extensively reviewing it at a networking conference visited by practitioners interested in participating in the project and researchers in the field of substance use prevention and treatment. This process further enhanced the foundation of the programme with regard to content and feasibility. In the third step, we conducted and closely monitored a pilot trial of the programme in one of the participating centres, after which further amendments were made.

The resulting detailed manual includes nine weekly 90-minute modules for the children as well as two optional parent sessions. Even though the modules build on one another, each module is also a closed unit with a specific theme: Module 1 - getting to know each other, module 2 - self-worth: how I feel about myself, module 3 - alcohol and/or drug problems in my family, module 4 - knowledge: what I need to know about drugs and addiction, module 5 - handling difficult emotions, module 6 - self-efficacy: what I can do to solve problems, module 7 - learning new patterns of behaviour in my family, module 8 - what I can do to find help and support, module 9 - a positive good-bye. The themes are delivered in an interactive and age-based way, with a large percentage of exercise and role-play. With regard to structure, special attention is paid to a recurring structure of the sessions and to small rituals, both of which children often lack in a substance-affected home [50]. Each session follows the same structure, beginning with an exchange on how they feel today, followed by discussing the "homework" from the last session, then introducing the new topic of the day and working on this with a variety of the didactics already mentioned. In between learning activities, there also are pure "fun-and-play" activities such as songs or creative exercises. Each session is finished off by a relaxation exercise.

The two parent sessions can be attended independently, taking into account that in volatile families like those at hand programme providers cannot assume that parents will come to both, or even one, of the sessions. The content of the first session, conducted at the beginning of the programme, is to inform parents about the programme their children will be attending and about risk and protective factors children face when growing up in a substance-affected environment. Also, parents share hopes they have for living together with their children and are encouraged regarding parenting skills and their importance for their children. The content of the second session, conducted at the end of the programme, is to inform parents about how the programme went from the trainer perspective, to answer questions about issues that may have come up at home in the course of the programme and to sensitize parents for the needs of children in substance-affected families and how caregivers may be empowered in the future. Also parents are motivated to seek and accept further support in their parenting role. The manual provides for parent questions, group discussion and practical exercises in both sessions.

For the control condition, we developed a similarly standardised manual for a play group named "Bouncy Castle". Here the children are engaged in "fun-and-play" activities only, such as drawing, crafts, exercise, and games for the same amount of time that is allotted to the TRAMPOLINE programme. We described the group as equally attractive to parents and children to minimize recruitment problems resulting from the randomisation. Besides, having children not only on wait-lists but in a regular intervention gives practitioners a means of possible crisis intervention, an option that many participating centres asked for out of ethical considerations. Trainers are requested to refrain from initiating the discussion of addiction-related topics in the group and to deal with them as briefly as ethically possible if they should come up. Parents in the control condition do not receive a structured intervention, although participating facilities are encouraged to inform them about the programme as well.

### Cooperating centres and participants

Eligible institutions for delivering the interventions, i.e. experienced in dealing with the clientele at hand, were identified by systematically scanning comprehensive help provision and therapy guides, using existing online and offline lists and conducting an internet search. We also gathered data on their relevant activities in the area by sending out a questionnaire on their work with children from substance-affected families [41]. All participating centres signed cooperation contracts before delivering the programme and received financial incentives for their participation. As a number of facilities agreed to cooperate, but could not recruit enough participants to conduct the intervention, several efforts had to be made during the entire field phase to engage and train new cooperation partners. In the end, 27 cooperating out-patient counselling centres participated in the project. Centres were almost uniformly distributed across the country with at least one participating centre in nearly every federal state. Most of the centres were addiction aid facilities and only some were youth and family support services or other groups. Participating facilities recruit participants using their existing recruitment strategies as well as recruitment material provided by the research team (flyers, webpage, posters, newspaper advertisements). They conduct intake talks with children and parents, inform them about the content and goal of the programme and screen participating children for eligibility. Inclusion criteria for children are: (1) age at beginning of the programme between 8 and 12 years, in exceptions after careful screening between 7 and 13 years if developmental phase fits (2) current or recent (within the last year) substance misuse or dependency in at least one parent (3) children either live with the substance-affected parent or have regular contact with him or her (4) sufficient mastery of the German language both in children and parents to participate in assessments (5) informed written consent of the parents and the children. Children are excluded from the study if (1) they are diagnosed with or suspected of foetal alcohol spectrum disorder by recruiting staff due to extreme behavioral difficulties or if (2) they have received any kind of addiction-specific treatment relevant to the study goals up to six months prior to the intervention. Participating children and parents do not receive financial incentives for participating in the intervention, but they do receive a small compensation for participating in data collection (10€ per parent and questionnaire, a small present worth 2-3€ per child and interview).

### Data collection

Data are gathered at three points: At baseline, i.e. two weeks prior to intervention (t0), shortly after the intervention (t1) and six months after the intervention (t2). Data from the third measurement point is collected until the end of January 2012. To address the expected parental concerns about anonymity mentioned above and to avoid bias, we trained bachelor-level students of social and public health science from all over Germany to gather data. The students visited the families at all three measurement points and interviewed the children while providing parents with paper-and-pencil questionnaires. Interviewers were extensively trained in the specific challenges of surveying substance-affected families and are closely supervised to deal with problems that may come up. For example, even though a mother had been informed that this was a group programme on drug use and addiction, she refused to let an interviewer survey her child on this topic since she had not "told him yet". In cases like this, the interviewer called the researchers for immediate guidance. With regard to measurements, we used age-adequate standardised instruments, where possible, to assess our outcome variables. Children report on socio-demographic data, current parental substance use, stress level and coping strategies, self-worth, self-efficacy and satisfaction with the intervention, relationship quality with the parent, addiction-related knowledge and health-related quality of life. Parents (ideally both) or primary caregivers report on psychological stress, their own substance use, relationship quality with the child, sensitivity for children's needs, assertiveness regarding their own parenting competence, parenting style, child coping strategies and child behavior. An overview of measurements is given in Table 1. Interviewers are blind to condition at baseline and at t1, but not at t2 due to the fact that only the parents in the intervention condition participate in group sessions and are asked about them in t2.

**Table 1:** Measures for children and parents

Target variable - CHILD	Measure
*Socio-demographic characteristics*	

*Self-concept/self-worth*	SPPC - Self Perception Profile for Children (German version) [51,52]

*Physical stress symptoms*	SSK - Fragebogen zur Erhebung von Stresserleben & Stressbewältigung im Kindesalter [53] Questionnaire for perception of stress and stress management in childhood Subscale: physical stress symptoms

*Quality of relationship between parent and child*	Own development: questions on a thermometer-scale in regard to closeness vs. distance and harmony vs. conflict

*Parental drug and addiction problems*	CAST 6 - German Children of Alcoholics Screening Test [54,55]

*Knowledge on alcohol, drugs, and substance use problems*	Own development

*Stress and coping*	SSKJ 3-8 - Fragebogen zur Erhebung von Stresserleben & Stressbewältigung im Kindes- und Jugendalter [56] Questionnaire for perception of stress and stress management in childhood and adolescence

*Mental distress caused by parental substance use*	Own development

*Expectations of self-efficacy*	WIRKALL-r - Psychometrische Skala zur Allgemeinen Selbstwirksamkeitserwartung (SWE) - für Kinder adaptierte Version [57,58] Psychometric scale for general expectation of self-efficacy - adapted version for children Adaptation of a scale used for measuring self-efficacy in the family [59,60]

*Health-related quality of life*	KIDSCREEN-27 - Health related quality of life questionnaire for children and young people and their parents [61,62]

**Target variable - PARENT**	**Measure**

*Socio-demographic characteristics*	

*Psychological stress*	Symptom checklist SCL-27-plus [63]

*Parental substance use problems*	Alcohol Use Disorders Identification Test (AUDIT) [64,65]

*Parental use of further substance-related aid*	Own development

*Parental sensitivity for the effects of substance use problems on children and children's need*	Own development

*General parenting behaviour*	ZKE-E - Kurzfragebogen zur Erfassung elterlicher Erziehungshaltungen [66] Brief questionnaire for perception of child-raising behaviour (parent version)

*Quality of relationship between parents and child*	Own development: questions on a thermometer-scale in regard to closeness vs. distance and harmony vs. conflict

*Self-confidence in parenting skills*	Fragebogen zum Kompetenzgefühl von Eltern (FKE) [67] Questionnaire for parental feelings of competence

*Child behavioural difficulties*	SDQ-D-German (Strength and Difficulties Questionnaire, German version) [68,69]

*Stress management strategies of* *the child*	SSKJ 3-8 - Fragebogen zur Erhebung von Stresserleben & Stressbewältigung im Kindes- und Jugendalter [56] Questionnaire for perception of stress and stress management in childhood and adolescence

Besides gathering outcome evaluation data on the effectiveness of the TRAMPOLINE programme, process evaluation data is also collected. Course instructors in the participating centres complete an extensive documentation on relevant variables (adherence, interaction with the group, possible disturbances) after each child and parent session. Also, the instructors are required to conduct visual recordings of two out of the nine child sessions chosen by the research team. Children and parents evaluate each session they participate in directly after the session is over. In addition, course instructors and institute managers complete questionnaires on relevant characteristics such as structural data on their institute or professional background. These data will be used to analyse the quality of manual adherence and to identify possible influencing factors such as instructor qualification or group atmosphere.

### Statistical analyses

The sample size for our study is based on a power analysis for detecting small effect sizes. In general, existing literature shows that medium effect sizes can occur, but these studies utilise wait-list controls so that effects may be stronger than in the study at hand. When using a two-sided test at alpha = 0.05 and a power of (1-beta) = 0.80, a total sample of at least N = 200 is required [70]. This takes a worst case scenario of 30% loss-to-follow-up after randomisation into account. Data will be analysed in a 2 × 2 ANCOVA design and in accordance with the per-protocol principle, since we are primarily interested in the effect of the programme on those children that actually participated in the programme from start to finish. In addition, in secondary analyses we will examine the data following the intention-to-treat principle to identify possible effects of attrition. A dropout analysis will be conducted. Missing data will be imputed. The ANCOVA approach will be employed for baseline-adjusted analyses. For computations, the SPSS standards software will be used.

### Quality assurance

Whenever possible, standardised instruments are used. All instruments were tested during the pilot of the intervention and experience from these interviews conducted by the researchers was passed on to the student interviewers in a one-day training session. Interviewers received a detailed guide for data collection and are closely monitored throughout the data collection. Cooperating centres received detailed study guidelines, informal information via telephone, recruitment material and the manuals themselves. The intervention in both conditions is delivered by expert staff in the cooperating institutions, mainly social workers, pedagoges, and psychologists often with additional therapeutic or counselling qualifications. A prerequisite for becoming a trainer is experience in working with substance-affected families and experience in conducting group formats. Trainers received an intensive one-day training with regard to delivering the intervention and are advised to keep close contact with the research group. At least two trainers are trained per cooperating centre to ensure a stand-in solution in case of illness or absence. Researchers conduct regular supervisory phone calls to ensure ongoing communication about the intervention and its delivery. The satisfaction of trainers and participants is assessed at the end of each session and will be analysed as part of the process evaluation.

### Ethical considerations

Participating centres, all participants and their parents received detailed information on the study goals, procedures, analyses and data reporting prior to participation. Parents and children need to give written consent to be able to participate in the study. Participants in the control condition are offered addiction-specific counselling (either in form of the group programme TRAMPOLINE or other services offered by participating facilities) after data gathering is completed. Participating families are encouraged to call the research group if they have questions or problems arise. All procedures are approved by the ethics committee of the Chamber of Physicians in all federal states of Germany where participating centres are located.

## Discussion

There is a substantial need for evidence-based programmes targeting children from substance-affected families. In Germany, the research project TRAMPOLINE presented in this study protocol is the first to develop and evaluate such a programme. It is hypothesised that the addiction-specific group programme TRAMPOLINE will elicit superior effects in participating children as compared with participants in an addiction-unrelated play group on several relevant indicators such as coping skills or psychological distress. By working together with many different institutions, we also hope to gather knowledge on the implementation of such a programme, especially concerning recruitment challenges and the integration of such a programme into the standard processes of outpatient counselling centres. The study and its intervention have several strengths: First, the intervention is carefully planned and tailored to the needs of children from substance-affected families, taking into account both theoretical foundations and experience of practitioners in the field. Second, the intervention is designed to be delivered by non-therapists and is easy to deliver due to the manual and to its modular structure. Thus, we view it as a pragmatic, low-threshold programme that can be disseminated widely into different contexts. Third, there is a strict separation between programme deliverers and programme evaluators so that bias is reduced. A further reduction of bias is achieved by gathering not only child self-report data, but also the caregivers' view on children's development. Fourth, in conducting a 6-month follow-up, we are able to detect sleeper effects and test the stability of effects uncovered in the post-measurement. Fifth: we conduct a rigorous evaluation by comparing the effects of the programme with an intervention of similar dosage, but without addiction-specific content.

The limitations of the study must also be mentioned: Due to the volatile nature of the families we target, it may be difficult to motivate them to participate in follow-up assessments. Also, many substance-affected parents will not let their children participate in the programme, thus creating a selection effect in the sample from the start. Moreover, we view the age range of the participating children as substantial and it must be seen if the programme will be beneficial for all age groups. For example, some exercises require writing and reading skills, and trainers have reported that especially younger children still need quite a lot of help in this area. Furthermore, it would be desirable to compare the effects of the programme with a naturalistic sample of children from substance-affected families not receiving any intervention. It was attempted to recruit this sample, but it quickly became clear that this recruitment challenge would exceed our resources. We also expect that attrition during the interventions will be substantial, because families view a weekly participation of their children in such a programme as a high demand on their daily routine. We try to handle this problem by encouraging our cooperating centres to contact the families if a child does not show up for a session and to support the families in any way they can regarding transportation and logistics. The reasons for dropouts are to be documented and will be analysed within the process evaluation. We hope to find out whether dropouts are due more to reasons such as time constraints in the family or rather to dissatisfaction with aspects of the programme or both. This analysis can possibly provide insights into the acceptance of the programme.

### Recruitment issues

The largest feasibility issue for our project was recruitment with the recruitment challenges described above. Since our programme is based on voluntary participation, parents' consent is needed. This, in turn, requires substance-affected parents to have insight into their own substance problems, know about services such as TRAMPOLINE and to be willing to overcome their inhibitions for confronting their children with the topic of addiction. Thus, even though a large group of children would be eligible for the programme, only a small percentage is accessible to us. In addition, the methodological requirement that no children be included that already receive addiction-specific TAU means that cooperating centres cannot let their "regulars" participate in the groups. Another factor that intensifies the problem is that professionals in Germany are not used to working with programmes of a comparably short duration, but usually work with families over a very long period of time, meaning months or even years. Since this requires a lot of resources, they are generally interested in shorter programmes such as TRAMPOLINE, at the same time, they must now persuade families to start at a given point in time and continue participating for nine weeks instead of inviting children into open groups that run weekly and are ongoing. We have approached these issues by providing cooperating facilities with a detailed guide specifying different possible recruitment strategies and also offering consultation on recruitment by the research team. We have also allotted time in the training sessions for exchanging knowledge on possible recruitment channels among the facilities. These are the recommendations we passed on to the recruiters in participating facilities: (1) Parents have often already shown up at some point in the health system. Therefore: extend the professional network of your institute as far as possible, inform different institutions such as medical practices, counselling centres, self-help groups, youth clubs about the programme (in as much detail as possible) and remind them of it in regular intervals. Use informal contacts as well as formal ways of informing about the project. (2) Conduct low-threshold information evenings about TRAMPOLINE in your institution. (3) Use other social meeting points in the living area of underprivileged families as places for information distribution. (4) Train school teachers so that they can identify children from substance-affected families and approach the children and their family members about possible participation. (5) Use media such as webpages or newspapers for publicity. Some newspapers will not only print an advertisment for the programme, but feature an article about the institution and its new programme. (6) Try to find cost-effective solutions for families' problems with logistics, such as a pick-up- and bring-home service. (7) Discuss recruitment options and experiences with other cooperating institutions to generate new ideas for yourself. At the same time we informed all ministries of the federal states of Germany about the participating centres and the programme and requested that they distribute the information to organisations in their network, for instance to federal state physicians' chambers. Furthermore, we provided support material for recruiters such as checklists for intake conversations, e.g., a list of arguments to answer possible concerns regarding randomisation, videotaping sessions and other anonymity issues. Finally, we installed a regular newsletter in which we inform the cooperating centres about what is going on in other centres and in which we encourage exchanging experience with the programme among practitioners.

## Conclusion

Implementation issues notwithstanding, our study provides the unique opportunity to develop and test a structured intervention for a target group in need, i.e. children from substance-affected families. It also adds to the quality of services for these families by building a close network among institutions working in this area within the project. This network will remain after the project is finalized and provides a solid foundation for the future dissemination of the TRAMPOLINE programme.

## Competing interests

The authors declare that they have no competing interests.

## Authors' contributions

SB drafted the manuscript. IS, MK and RT obtained funding. AW and SB are coordinating the study. SB, DM, SR and IS participated substantially in designing the intervention and study methodology. LW is responsible for data analysis. MK and RT were involved in all parts of the article as general supervisors of the research groups. All authors have read and approved the final manuscript.

## Authors' information

The authors of this paper work at the two partner institutes currently conducting this national trial of a community-based intervention for children from substance-affected families in Germany http://www.projekt-trampolin.de. For more information on our research, see http://www.dzskj.de, http://www.disup.de.

## Pre-publication history

The pre-publication history for this paper can be accessed here:

http://www.biomedcentral.com/1471-2458/12/223/prepub
